# Induction of Apoptosis, Inhibition of MCL-1, and VEGF-A Expression Are Associated with the Anti-Cancer Efficacy of Magnolol Combined with Regorafenib in Hepatocellular Carcinoma

**DOI:** 10.3390/cancers13092066

**Published:** 2021-04-25

**Authors:** Cheng-Hsien Chen, Fei-Ting Hsu, Wei-Lung Chen, Jiann-Hwa Chen

**Affiliations:** 1 Surgical Department of Show Chwan Memorial Hospital, Changhua 500, Taiwan; aDS406@show.org.tw; 2 Department of Biological Science and Technology, China Medical University, Taichung 406, Taiwan; sakiro920@mail.cmu.edu.tw; 3 Department of Emergency Medicine, Cathay General Hospital, Taipei 106, Taiwan; cgh01579@cgh.org.tw; 4 School of Medicine, Fu Jen Catholic University, New Taipei City 242, Taiwan

**Keywords:** magnolol, regorafenib, apoptosis, MCL-1, VEGF-A, hepatocellular carcinoma

## Abstract

**Simple Summary:**

The synergistic inhibition of hepatocellular carcinoma growth was induced by administering magnolol together with regorafenib, instead of each treatment individually. Hepatocellular carcinoma (HCC) cells were sensitized to regorafenib through the inhibition of the expression of both vascular endothelial growth factor A (VEGF-A) and myeloid cell leukemia 1 (MCL-1) by siRNA. Moreover, the regorafenib-induced suppression of VEGF-A and MCL-1 at the protein level was enhanced by magnolol. Extrinsic (expression of FAS, FAS-L, and cleaved-caspase-8) and intrinsic apoptotic signaling (ROS production, the accumulation of Ca^2+^, the loss of △ψm, and the nuclear translocation of AIF), and DNA damage were all effectively increased by regorafenib combined with magnolol. In addition, a superior inhibition of metastasis was triggered by the combination of regorafenib and magnolol. In sum, the enhancement of apoptosis induction and the suppression of the expression of VEGF-A and MCL-1 were associated with the anti-cancer efficacy of magnolol combined with regorafenib in HCC.

**Abstract:**

While regorafenib was approved for the treatment of advanced HCC in 2017, with a partial response and survival benefit; other combination agents to facilitate the efficacy of regorafenib still need to be explored. Magnolol is a potential natural anti-tumor compound for many types of cancers. Combination indexes calculated on the basis of both in vitro and in vivo models have indicated a synergistic effect of the combination of regorafenib and magnolol. The overexpression of the VEGF-A protein significantly diminished regorafenib’s inhibition of cell viability, while the transient knockdown of VEGF-A by siRNA effectively sensitized HCC cells to regorafenib. In addition, the inhibition of MCL-1 by siRNA combined with regorafenib allowed for a significantly greater inhibition of cell growth, compared to regorafenib alone. A lower protein expression level for VEGF-A and MCL-1 was found for the combination treatment of HCC in vitro and in vivo. A superior metastasis inhibition was also found in the combination group, as compared to the single-treatment groups, using a transwell assay, wound healing assay, and Western blotting. The caspase-dependent and -independent and DNA damage effects, as determined by flow cytometry and a comet assay, were increased by the combination therapy. Taken together, magnolol sensitized HCC to regorafenib, which was correlated with the reduction of VEGF-A and MCL-1 and the induction of apoptosis.

## 1. Introduction

Regorafenib, an oral multi-kinase inhibitor derived from sorafenib, is used with sorafenib for the treatment of metastatic colorectal cancer (mCRC), advanced gastrointestinal stromal tumors, and hepatocellular carcinoma (HCC), after they have progressed [1,2]. Regorafenib alleviates tumor growth, angiogenesis, and metastasis by inactivating angiogenic and oncogenic kinases, such as the vascular endothelial growth factor (VEGF), platelet-derived growth factor (PDGF) receptor, Raf, and mast/stem cell growth factor receptor (c-KIT) [3,4]. Preclinical and clinical studies presented that natural compounds extracted from medicinal plants enhanced the therapeutic efficacy of regorafenib in HCC and mCRC [5,6].

Chlorogenic acid (CGA), a polyphenol isolated from many plants, has been indicated to potentiate the anti-growth effect of regorafenib via the blockage of mitogen-activated protein kinase (MAPK) and phosphatidylinositol-3-kinase (PI3K)/AKT signaling in HCC cells. In addition, CGA elicited regorafenib-induced apoptosis through the intrinsic (mitochondrial) pathway [5]. Silybin, a functional compound presented in the seeds of Milk Thistle, has been demonstrated to reduce liver damage induced by regorafenib and augment the clinical efficacy of regorafenib in patients with mCRC. The suppression of the AKT/mechanistic target of the rapamycin (mTOR) pathway was associated with the synergistic anti-proliferative and apoptotic effect of silybin in combination with regorafenib in CRC cells [6].

Magnolol, a multifunctional ingredient of the medicinal plant Magnolia officinalis, has been shown to enhance anti-growth, anti-angiogenic, and anti-metastatic effects through the induction of cell cycle arrest and apoptosis and the suppression of critical oncogenic pathways involved in tumor progression in various cancers [7,8,9]. Cells and animal models demonstrated that magnolol, as a sorafenib sensitizer, enhanced the anti-HCC efficacy of sorafenib. Magnolol significantly boosted the sorafenib-induced inhibition of growth and invasion potential through the suppression of AKT signaling [10]. However, how magnolol sensitizes HCC cells to regorafenib has not yet been elucidated. Therefore, the major goal of the present study was to investigate the anti-cancer efficacy and mechanism of magnolol combined with regorafenib in HCC cells in vitro and in vivo.

## 2. Materials and Methods

### 2.1. Chemicals and Reagents

Magnolol, regorafenib, 3-(4,5-dimethylthiazol-2-yl)-2,5-diphenyltetrazolium bromide (MTT), and dimethyl sulfoxide (DMSO) were purchased from Sigma Chemical Co. (St. Louis, MO, USA). VEGF-A recombinant protein was purchased from Elabscience (cat: PKSH033475, Houston, TX, USA).

### 2.2. Cell Cultures, Viability Analysis, and Combination Index Analysis

Hep3B and SK-Hep1 cells were maintained in Dulbecco’s Modified Eagle Medium (DMEM) with high glucose, 10% fetal bovine serum, 2 mM of L-glutamine, 100 U/mL of penicillin, and 100 mg/mL of streptomycin (Thermo Fisher Scientific, Fremont, CA, USA). The cells were placed in a humidified incubator at 37 °C with 5% CO_2_. For viability, Hep3B and SK-Hep1 cells were seeded in 96-well plates (5000 cells/well) overnight and treated with 0–20 μM regorafenib, 0–100 μM magnolol, and a combination of both. MTT (MTT 3-(4,5-dimethylthiazol-2-yl)-2,5-diphenyltetrazolium bromide) assays were then performed to identify the cell viability. Combination index (CI) analysis was followed by Chou’s studies [11]. If the combination index was smaller than 1, this can be defined as a synergistic effect resulting from the combination itself, as opposed to a single treatment [10].

### 2.3. Transfection Procedure of siMCL-1 and siVEGF-A

Hep3B and SK-Hep1 cells (2 × 10^5^) were seeded in 12-well plates overnight. The cells were then transfected with siRNA UNIVERAL control, siRNA VEGF-A (Gene ID: 7422), and siRNA MCL-1 (Gene ID: 4170), followed by the DharmaFECT™ transfection—siRNA transfection protocol (Dharmacon siGENOME, Level Biotechnology, Inc., Taipei, Taiwan). In brief, 5 μL of siRNA (5 μM) solution was added to 95 μL of a serum-free medium. In addition, 5 μL of the DharmaFECT reagent was also added to 95 μL of the serum-free medium. Then, the two tubes were gently mixed by pipetting and incubation at 25 °C for 20 min and added to an antibiotic-free complete medium to obtain a total volume of 1 mL of a transfection medium. Finally, the 12-well medium was replaced with 1 mL of the transfection medium and incubated at 37 °C for 72 h. The transfected cells were then subjected to an MTT assay and Western blotting, after combining them with 30–50 ng/mL of VEGF-A recombinant protein or regorafenib 0–20 μM (or not) for 48 h of treatment.

### 2.4. Immunofluorescence Staining

Hep3B and SK-Hep1 cells (2 × 10^5^) were seeded in 4-chamber slices and incubated with 50 μM magnolol, 10 μM regorafenib, and a combination for 48 h. Then, the cells were fixed with 4% formaldehyde for 15 min, permeabilized with 0.1% Triton X-100/PBS for 10 min and blocked with 1% bovine serum albumin (BSA) in phosphate-buffered saline (PBS). The fixed cells were incubated with primary antibodies against AIF (Elabscience, D39D2 XP^®^ Rabbit mAb #5318, 1:300 dilutions) overnight. The slides were then rinsed with PBST (0.01% Tween 20) and exposed to secondary antibody (FITC-conjugated goat anti-rabbit IgG at 1:300 dilution) at 37 °C for 1 hr. Finally, the slides were photographed using a fluorescence microscope (Axio Imager 2, Zeiss, NY, USA) at 200× magnification [12].

### 2.5. Comet Assay 

The DNA damage was measured using a comet assay. The comet assay was followed by the method published by Singh et al. with slight modifications [13]. Hep3B and SK-Hep1 cells were seeded in 12-well plates, with 2 × 10^5^/well, overnight and then incubated with 50 μM magnolol, 10 μM regorafenib, a combination, or 0.1% H_2_O_2_ for 48 h. A caspase inhibitor, ZVAD (50 μM), was treated 30 min before combining with magnolol and regorafenib. Conventional microscope slides were coated with 85 μL of 0.5% normal-melting-point agarose (NMP, Sigma-Aldrich) and 0.5% low-melting-point agarose (LMP) in PBS (pH 7.4) and allowed to dry on a flat platform at 25 °C. Subsequently, 10 μL of cell suspension (2.5 × 10^5^ cells/mL) was gently mixed with 75 μL of 0.5% (*w*/*v*) LMP in PBS (pH 7.4), rapidly layered onto slides pre-coated with mixtures of 0.5% NMP and 0.5% LMP, and covered with a coverslip and maintained at 4 °C. After 5 min, the coverslip was removed, and the cells were immersed in a lysis buffer (2500 mM of NaCl, 100 mM Na_2_EDTA, 10 mM Tris and 1% (*v*/*v*) of Triton X-100 at pH 10) at 4°C for 1 h. The slices were washed twice and transferred to a horizontal electrophoresis tank with an alkaline buffer (300 mM NaOH and 1 mM Na_2_EDTA at pH 13) at 4 °C for 20 min. Thereafter, electrophoresis was conducted at 30 V and 300 mA for another 20 min. After running the procedure, the slices were then dipped in a cold neutralizing buffer (400 mM of Tris-HCl, pH 7.5) at 4 °C for 15 min, dried in methanol for 5 min, and stained with 50 μL of Propidium Iodide (PI, 2.5 μg/mL). Finally, the slides were photographed using a fluorescence microscope (Nikon ECLIPSE Ti-U, Minato City, Tokyo, Japan) at 200× magnification and quantified by image J version 1.50 (National Institutes of Health, Bethesda, MD, USA) using the OpenComet v1.3.1 tool box [14].

### 2.6. Western Blotting

The Hep3B and SK-Hep1 cells (2 × 10^5^) were seeded in a 10-cm dish and incubated with 50 μM magnolol, 10 μM regorafenib, and a combination for 48 h. Forty micrograms of protein were separated by 8–12% SDS-PAGE and transferred onto a PVDF membrane [15,16]. Primary antibodies against CyclinD1, X-linked inhibitor of apoptosis (XIAP), cellular FLICE (FADD-like IL-1β-converting enzyme)-inhibitory protein (c-FLIP), MCL-1, matrix metallopeptidase 9 (MMP9), VEGF-A, mediator of DNA damage checkpoint 1 (MDC-1), Bcl-2-associated X protein (BAX), Bcl-2 homologous antagonist killer (BAK), FAS, FAS-L, cleaved-caspase-3, -8, and -9, cleaved Poly [ADP-ribose] polymerase 1 (PARP-1), and β-actin (Elabscience) were used for the conjugation overnight, followed by incubation with secondary antibodies. The protein signal was detected using an Immobilon Western Chemiluminescent HRP Substrate kit (EMD Millipore) and captured with a chemiluminescent imaging system (ChemiDoc-It 515, UVP, Upland, CA, USA).

### 2.7. Flow Cytometry Analysis

Hep3B and SK-Hep1 cells (5 × 10^5^) were seeded in 6-well plates and incubated with 50 μM magnolol, 10 μM regorafenib, and a combination for 48 h. ZVAD was added for 30 min before magnolol combined with regorafenib for 48 h. The cells were then harvested for the staining of different reagents, including Annexin-V/PI, cleaved-caspase-3 (1 μL, fluorescein isothiocyanate-Asp(OCH3)-Glu(OCH3)-Val-Asp(OCH3)-fluoromethyl ketone (FITC-DEVD-FMK)), cleaved-PARP-1, FAS-FITC (1 μL), FAS-L-PE (1 μL), cleaved-caspase-8 (1 μL, sulforhodamine-Ile-Glu-Thr-Asp-fluoromethyl ketone (Red-IETDFMK), cleaved-caspase-9 (1 μL FITC-Leu-Glu-His-Asp-fluoromethyl ketone (FITC-LEHD-FMK)), DCFH-DA (500 μL at 10 μM) for ROS, DIOC6 (4 μmol/L) for Dy loss, and Fluo-3/AM (2.5 μg/mL) for Ca^2+^. For subG1 analysis, the harvested cells were fixed by 70% ethanol overnight at −20 °C and stained by a PI/RNase solution (cat: 550625, BD Biosciences). The fluoresce signal from the cells was detected and quantified by NovoCyte flow cytometry and the NovoExpress^®^ software (Agilent Technologies Inc., Santa Clara, CA, USA) [15,17].

### 2.8. Wound Healing Assay

The Hep3B and SK-Hep1 cells (5 × 10^5^) were seeded in 6-well plates overnight and treated with 50 μM magnolol, 10 μM regorafenib, and a combination for 48 h. The cells (1 × 10^5^/per well) were then seeded in 6 wells with ibidi culture-inserts (cat: 80241, ibidi GmbH, Gräfelfing, Germany) overnight, allowing for cell migration. The cell migration pattern was photographed using a Nikon ECLIPSE Ti-U microscope (Tokyo, Japan) at 0, 12, and 24 h after migration [15].

### 2.9. Invasion and Migration Assay

The Hep3B and SK-Hep1 cells (5 × 10^5^) were seeded in 6-well plates overnight and treated with 50 μM magnolol, 10 μM regorafenib, and a combination for 48 h. The viable cells counted by trypan blue were then collected for transwell migration and invasion analysis, as previously described [17].

### 2.10. Animal Experiment

Six-week-old nude mice (BALB/cAnN.Cg—Foxn1nu/CrlNarl) were purchased from the National Laboratory Animal Centre (Taipei, Taiwan) and maintained in a pathogen-free animal center in China Medical University (CMU). The animal experiment was approved by the Animal Care and Use Committee in CMU and given approval number: CMU IACUC 2021-252. The Hep3B cells (1 × 10^7^/mice) were inoculated in the right flank of the mice for 10–14 days of growth. When the average tumor volume reached 100 mm^3^, the mice were separated into a non-treated control and three different treatment groups. The control group was treated with 0.1% DMSO in 100 μL of PBS per day by gavage. The magnolol and regorafenib groups were treated with 100 mg/kg of magnolol or 10 mg/kg of regorafenib in 100 μL of PBS per day by gavage. The combination group was treated with regorafenib combined with magnolol and dissolved in 100 μL of PBS/treat/gavage. The tumor volume, tumor weight, and body weight were measured once per five days by a caliper and digital scale. The mice were subjected to a micro-CT scan on day 0 and 21, as previously described [18].

### 2.11. Hematoxylin and Eosin (H&E) and Immunohistochemistry (IHC)

The tumors, kidneys, livers, and spleens of the mice were isolated on day 20, after the treatments. The organs (livers, spleens, and kidneys) of the mice were subjected to H&E staining by Bio-Check Laboratories Ltd. (New Taipei City, Taiwan), as previously described. Tumor tissue slices were used to identify the expression of CyclinD1, XIAP, C-FLIP, MCL-1, MMP9, VEGF-A, MDC-1, cleaved caspase-3, -8, and -9, Endonuclease G (EndoG), and apoptosis inducing factor mitochondria associated 1 (AIF) [19].

### 2.12. Statistical Analysis

The results are displayed as the mean ± SD. The statistical values were calculated by one-way ANOVA using the Newman–Keuls multi-comparison test. A *p*-value < 0.05 was defined as a significant difference between the control and treatment groups.

## 3. Results

### 3.1. Magnolol Effectively Induced the Cytotoxicity of Regorafenib in HCC Cells

**Figure 1:**
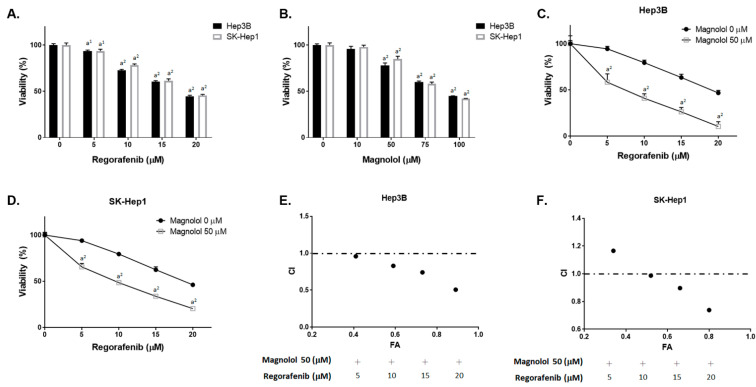

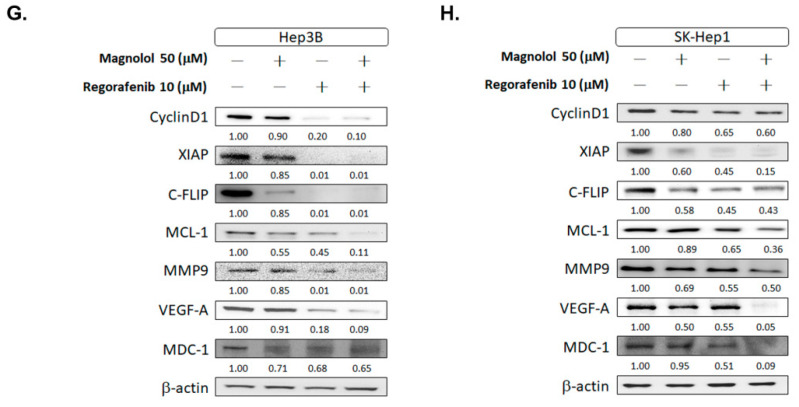
Cytotoxicity of regorafenib was triggered by magnolol. Hep3B and SK-Hep1 cells were treated with (**A**) 0–20 μM regorafenib, (**B**) 0–100 μM magnolol, or (**C**,**D**) 50 μM magnolol combined with 0–20 μM regorafenib for 48 h and assayed by the MTT assay. The combination index value for 50 μM magnolol combined with 0–20 μM regorafenib in (**E**) Hep3B and (**F**) SK-Hep1 cells is presented. The protein expression of CyclinD1, XIAP, C-FLIP, MCL-1, MMP-9, VEGF-A, and MDC-1 is presented after the treatments with magnolol, regorafenib, and a combination in (**G**) Hep3B and (**H**) SK-Hep1 cells. (a^1^ *p* < 0.05 and a^2^ *p* < 0.01 vs. 0 μM regorafenib or magnolol).

As illustrated in Figure 1A,B, the treatments with regorafenib and magnolol individually for 48 h may induce the cytotoxicity of Hep3B and SK-Hep1 cells. Additionally, as shown in Figure 1C,D, the cell viability was markedly decreased for magnolol combined with regorafenib. The CI value indicated that 50 μM magnolol combined with 10 μM regorafenib showed a synergistic toxicity efficacy in both the Hep3B and SK-Hep1 cells (Figure 1E,F). The CI value for different combination dosages is presented in Table 1. Thus, the CI value for 50 μM magnolol and 10 μM regorafenib were 0.89 and 0.93 in Hep3B and SK-Hep1 cells, respectively. Thus, above combination dosage was used for the validation of further experiments. In Figure 1G,H, various oncogenes involving proliferation (CyclinD1), anti-apoptosis (XIAP, C-FLIP, and MCL-1), DNA damage (MDC-1), and metastases (MMP-9 and VEGF-A) [20,21,22,23] were all effectively suppressed by regorafenib combined with magnolol. Full blot images are displayed in Figure S1. It is worth noting that the protein expression inhibition efficacy of VEGF-A and MCL-1 was especially found for the combination treatment. 

### 3.2. Inhibition of Both VEGF-A and MCL-1 Expression Sensitize HCC Cells to Regorafenib

**Figure 2:**
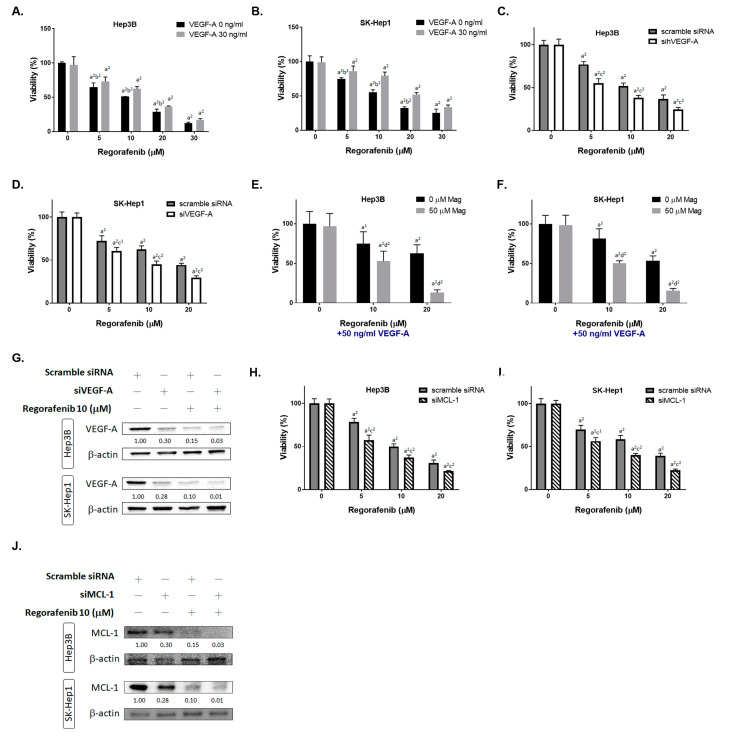
Inhibition of VEGF-A and MCL-1 may enhance the cytotoxicity of regorafenib. (**A**) Hep3B and (**B**) SK-Hep1 cells were treated with 0–20 μM regorafenib combined with 30 ng/mL of VEGF-A recombinant protein (or not) for 48 h, and the cell viability was measured via the MTT assay. (**C**) The Hep3B and (**D**) SK-Hep1 cells were transfected with siVEGF-A combined with 0–20 μM regorafenib (or not) for 48 h, and the cell viability was determined via the MTT assay. (**E**) The Hep3B and (**F**) SK-Hep1 cells were treated with 0–20 μM regorafenib, 50 ng/mL of VEGF-A recombinant protein, and 50 μM magnolol for 48 h, and the cell viability was determined via the MTT assay. (**G**) The VEGF-A Western blotting results in Hep3B and SK-Hep1 cells after being transfected with siVEGF-A combined with 0–20 μM regorafenib (or not) for 48 h. (**H**) The Hep3B and (**I**) SK-Hep1 cells were transfected with siMCL-1 combined with 0–20 μM regorafenib (or not) for 48 h, and the cell viability was determined via the MTT assay. (**J**) The MCL-1 Western blotting results in Hep3B and SK-Hep1 cells after transfection with siMCL-1 combined with 0–20 μM regorafenib (or not) for 48 h. (a^1^ *p* < 0.05 and a^2^ *p* < 0.01 vs. 0 μM regorafenib; b^1^ *p* < 0.05 and b^2^ *p* < 0.01 vs. 30 ng/mL VEGF-A; c^1^ *p* < 0.05 and c^2^ *p* < 0.01 vs. scramble siRNA; d^2^ *p* < 0.01 vs. 0 μM MAG).

To identify the role of VEGF-A in HCC cells, the cell viability of regorafenib was tested after being combined with the VEGF-A recombinant protein. As indicated in Figure 2A,B, the cytotoxicity induced by regorafenib was reversed by VEGF-A. On the contrary, the cytotoxicity of regorafenib was enhanced by VEGF-A silencing in both Hep3B and SK-Hep1 cells (Figure 2C,D). Moreover, magnolol can effectively reduce the cell viability of regorafenib, even with an additional exogenous VEGF-A administration. The results shown in Figure 2E,F indicate that magnolol may act as a VEGF-A inhibitor to induce the toxicity effect of regorafenib. Superior VEFG-A protein inhibition efficacy was found for siVEGF-A combined with regorafenib, as compared to regorafenib alone or siVEGF-A alone (Figure 2G). In addition, the inhibition of MCL-1 may also potentiate the cytotoxicity of regorafenib (Figure 2H,I). The protein expression of MCL-1 was significantly suppressed by siMCL-1 combined with regorafenib (Figure 2J). Full blot images are displayed in Figure S2. In sum, we suggest that the cytotoxicity enhancement of regorafenib by magnolol may be associated with VEGF-A and MCL-1 inhibition.

### 3.3. Magnolol Enhanced the Metastasis Inhibiton of Regorafenib in HCC Cells

**Figure 3:**
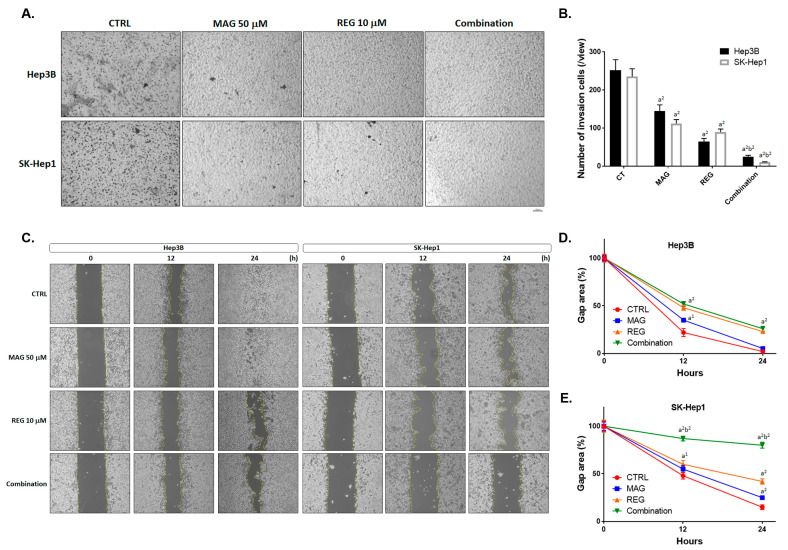
The metastasis inhibition effect of regorafenib was enhanced by magnolol. Hep3B and SK-Hep1 cells were treated with 10 μM regorafenib, 50 μM magnolol, and a combination for 48 h and subjected to a transwell invasion assay and wound healing assay. (**A**) A photograph of the invasion transwell and (**B**) the quantification results of the invasion cells. (**C**). A photograph of the migration cell pattern and the quantification results of the migrated gap area in (**D**) the Hep3B and (**E**) SK-Hep1 cells. (a^2^ *p* < 0.01 vs. CTRL; b^2^ *p* < 0.01 vs. 10 μM REG or 50 μM MAG; CTRL: Control, MAG: magnolol; REG: regorafenib).

According to Figure 1G,H, the metastasis-related proteins such as MMP-9 and VEGF-A were suppressed by regorafenib combined with magnolol. We then performed a transwell invasion assay and wound healing assay to confirm the enhancement of the metastasis-inhibition potential of regorafenib by magnolol. In Figure 3A,B, the number of invasion cells was significantly reduced in the combination group, as compared with the single-treatment groups. Additionally, the migration inhibition efficacy was superior in the combination group than in the single-treatment groups (Figure 3C). The gap area was relatively larger in the combination group, as compared to the single-treatment groups (Figure 3D,E). Taken together, a superior metastasis inhibition potential was found in the combination group, rather than the single-treatment groups.

### 3.4. Magnolol Induced the Apoptosis and DNA Damage Effect of Regorafenib in HCC Cells

**Figure 4:**
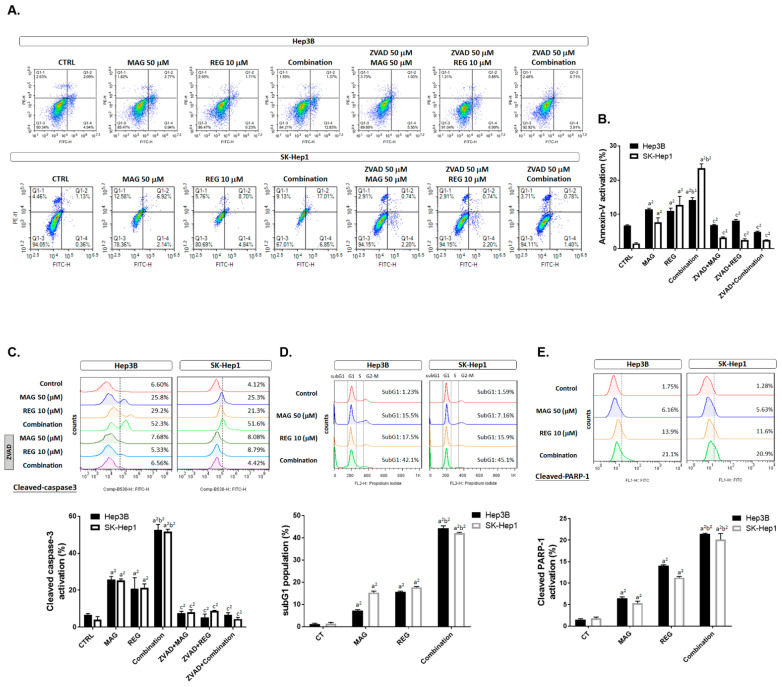

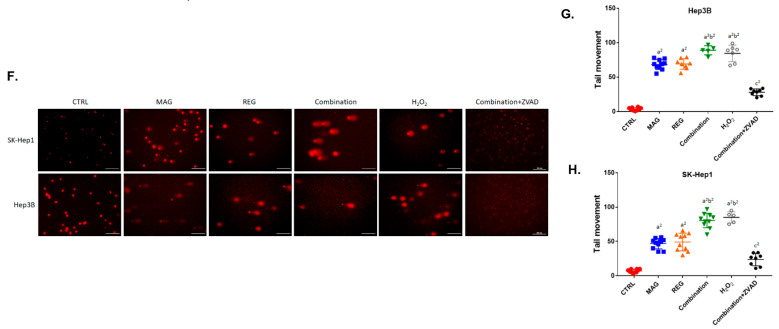
The apoptosis and DNA damage effect of regorafenib was enhanced by magnolol. Hep3B and SK-Hep1 cells were treated with 10 μM regorafenib, 50 μM magnolol, and a combination of both for 48 h, combined with ZVAD for 30 min, and assayed by flow cytometry with (**A**,**B**) annexin-V/PI staining and (**C**) cleaved caspase-3. After 48 h, the magnolol and regorafenib treatments were also performed with (**D**) PI staining and (**E**) cleaved PARP-1 staining, respectively. H_2_O_2_ was used as a positive control in the comet assay. The tail movement pattern of the Hep3B and SK-Hep1 cells were (**F**) photographed and (**G**,**H**) quantified. (a^2^ *p* < 0.01 vs. CTRL; b^2^ *p* < 0.01 vs. 10 μM REG or 50 μM MAG; c^2^ *p* < 0.01 vs. treatment groups without ZVAD; scale bar = 200 μm).

As shown in Figure 4A,B, the PI and annexin-V double-positive population (such double positivity is an apoptosis marker) was markedly increased to 25–30% in the combination group, compared to the single-treatment groups. Cleaved-caspase-3, an apoptosis marker, was also increased to 50–60% by regorafenib combined with magnolol (Figure 4C). In addition, to confirm that the apoptosis induction of magnolol combined regorafenib was mediated by caspase-dependent apoptosis, we added ZVAD (caspase inhibitor) to the magnolol, regorafenib, and combination treatments. As indicated in Figure 4A–C, the activation of annexin-V and cleaved caspase-3 by magnolol, regorafenib, and the combination treatments was diminished by an additional administration of ZVAD. Furthermore, another apoptosis marker, the subG1 population and cleaved PARP-1, were clearly increased by the combination treatment (Figure 4D,E). In Figure 4F, the DNA comet assay results indicated that the comet’s tail was increased in the combination group but reversed by ZVAD administration. The quantification results of the tail movement were increased in the Hep3B and SK-Hep1 cells in the combination group (Figure 4G,H). In sum, the apoptosis markers, annexin-V, cleaved caspase-3, subG1 population, cleaved PARP-1, and DNA damage, were all effectively increased by regorafenib combined with magnolol, as compared to the single therapies.

### 3.5. Magnolol Triggered the Caspase-Dependent and Caspase-Independent Apoptotic Effects of Regorafenib in HCC Cells

**Figure 5:**
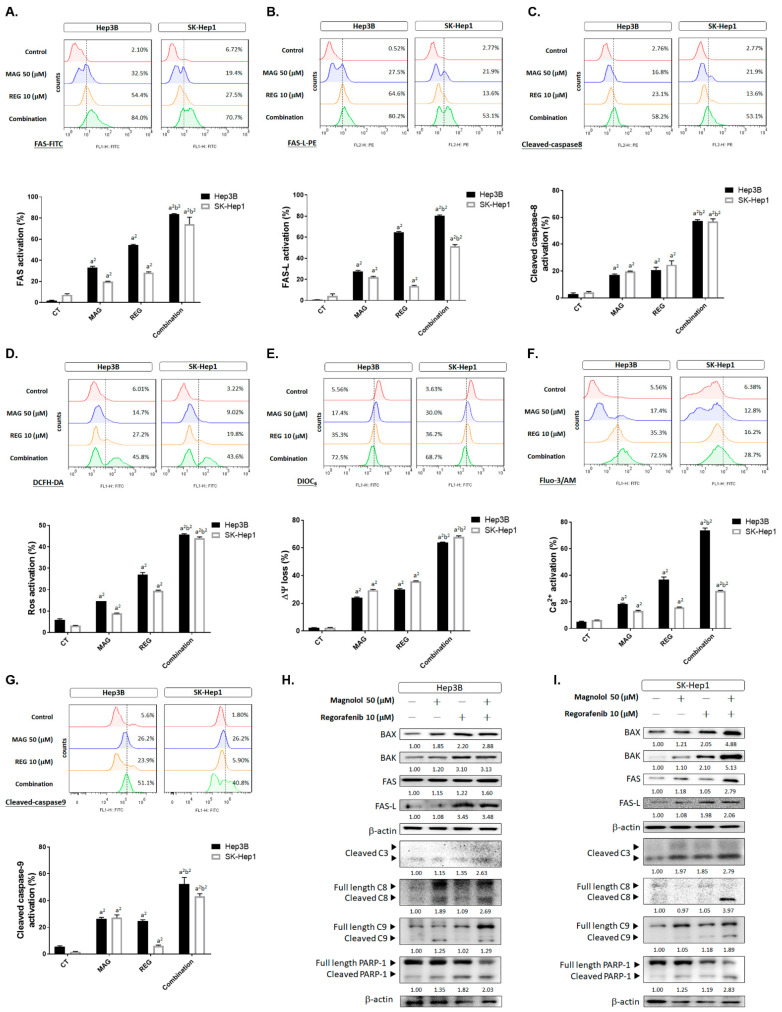

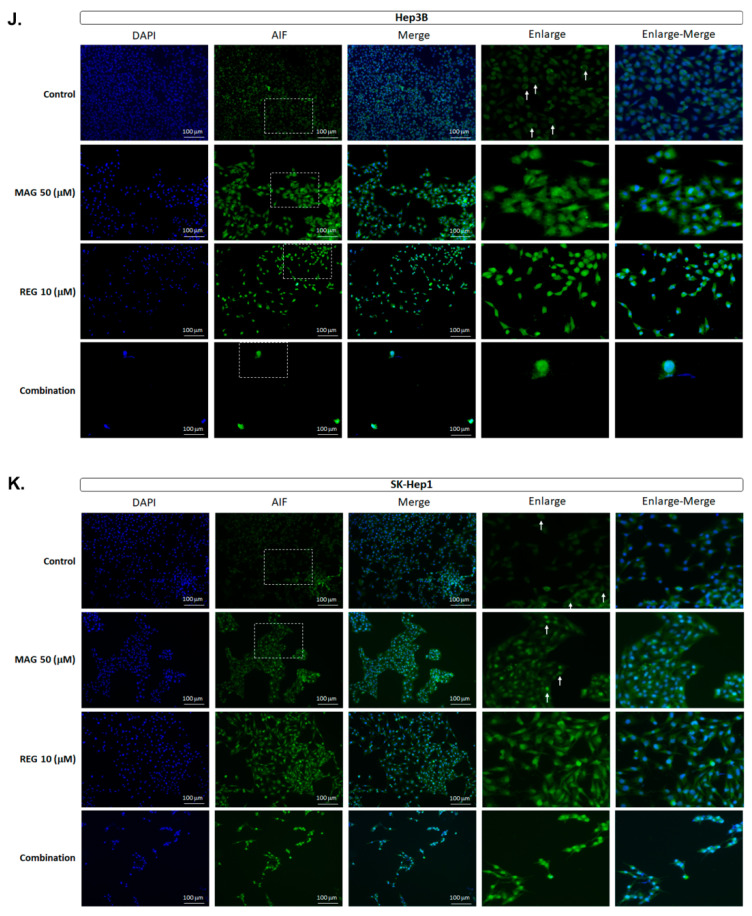
The caspase-dependent and -independent apoptotic effects of regorafenib were enhanced by magnolol. Hep3B and SK-Hep1 cells were treated with 10 μM regorafenib, 50 μM magnolol, and a combination for 48 h and assayed by flow cytometry using (**A**) FAS staining, (**B**). FAS-L staining, (**C**) cleaved caspase-8 staining, (**D**) DCFH-DA staining for ROS, (**E**) DIOC_6_ staining for ∆ψm, (**F**) Fluo-3/AM for Ca^2+^, and (**G**) cleaved caspase-9. The Western blotting of BAX, BAK, FAS, FAS-L, cleaved caspase-3 (cleaved C3), -8 (cleaved C8), -9 (cleaved C9), and cleaved PARP-1 in (**H**) the Hep3B and (**I**) SK-Hep1 cells. The IF staining of AIF in (**J**) Hep3B and (**K**) SK-Hep1 cells. (a^2^ *p* < 0.01 vs. CTRL; b^2^ *p* < 0.01 vs. 10 μM REG or 50 μM MAG; scale bar = 100 μm).

To further identify the apoptosis-inducing mechanism of regorafenib combined with magnolol, intrinsic and extrinsic apoptotic signaling was detected by flow cytometry. As shown in Figure 5A,B, the death receptor-dependent molecule FAS and its ligand (FAS-L) were increased by 50–80% in the combination group. We then identified whether, after FAS and FAS-L binding, caspase-8 was subsequently activated in the combination treatment group. The activation of cleaved-caspase-8 was increased to 60% in the combination group, as compared to the 20% induction in the single-treatment group (Figure 5C). Furthermore, the induction of reactive oxygen species was recognized through early stage mitochondrion-dependent apoptosis [24], which was also increased by the combination therapy (Figure 5D). The loss of both mitochondrial potential (△ψm) and Ca^2+^ was enhanced by regorafenib combined with magnolol (Figure 5E,F). Moreover, cleaved caspase-9 was effectively activated by the combination therapy (Figure 5G). Thereafter, we also investigated apoptosis-related proteins, such as BAX, BAK, FAS, FAS-L, cleaved caspase-3, -8, and -9, and cleaved PARP-1, using Western blotting to confirm the intrinsic and extrinsic apoptosis-inducing effect of magnolol and regorafenib. As illustrated in Figure 5H,I, the protein expression of BAX, BAK, FAS, FAS-L, cleaved caspase-3, -8, and -9, and cleaved PARP-1 was higher in the combination group than the single-treatment group. Full blot images are displayed in Figure S2. These results suggest that the apoptosis-promoting efficacy of regorafenib with magnolol was associated with the activation of intrinsic and extrinsic apoptosis pathways. Besides the caspase-dependent apoptosis of magnolol combined with regorafenib, we also investigated the caspase-independent apoptosis nucleus translocation of AIF. As shown in Figure 5J,K the nucleus translocation of AIF was effectively increased by regorafenib combined with magnolol.

### 3.6. Magnolol Enhanced the Anti-HCC Efficacy of Regorafenib in an Hep3B-Bearing Animal Model

**Figure 6:**
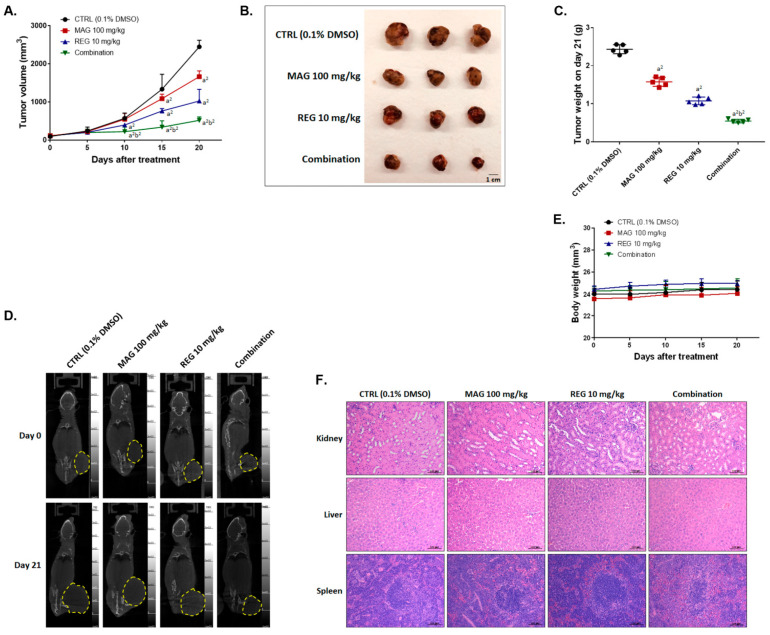

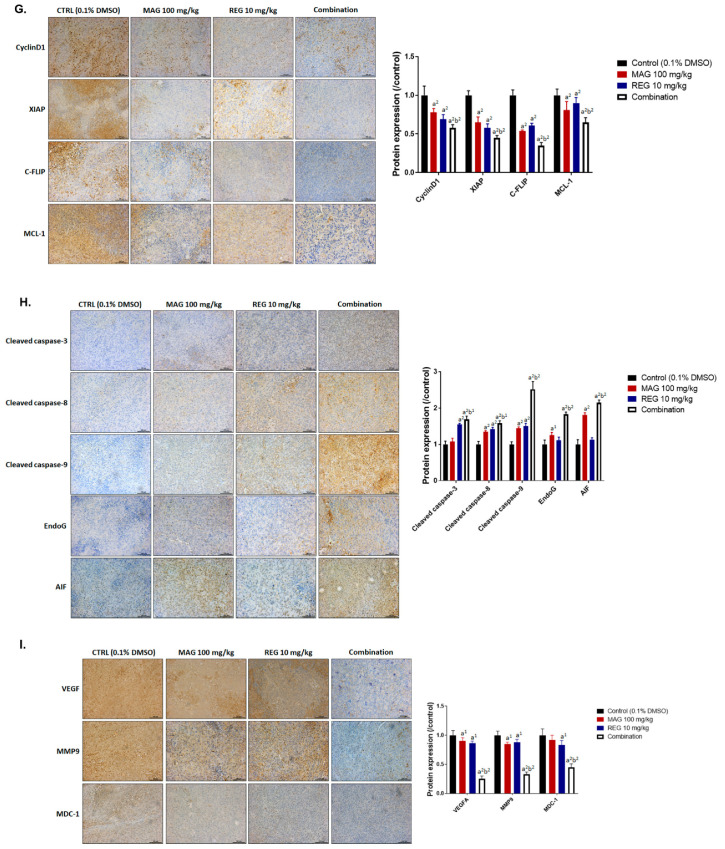
The therapeutic efficacy of regorafenib in Hep3B-bearing mice was enhanced by magnolol. (**A**) The tumor volume and (**B**) photographs and (**C**) weight are displayed after various treatments. (**D**) Micro-CT scan images, (**E**) mice body weight, and (**F**) an H&E stain of the liver, kidneys, and spleen are presented. IHC stain against (**G**) tumor progression, (**H**) apoptosis, (**I**) metastasis- and DNA repair-related antibodies are shown in the tumor tissue of each group. (a^1^ *p* < 0.05 and a^2^ *p* < 0.01 vs. CTRL; b^2^ *p* < 0.01 vs. 10 μM REG or 50 μM MAG; scale bar = 100 μm).

A Hep3B-bearing animal model was established and used to identify whether magnolol promotes the anti-HCC therapeutic efficacy of regorafenib. The tumor volume of Hep3B-bearing mice was significant decreased in the combination group and reached a significant difference after 10 days of treatment, as compared to the single-treatment group (Figure 6A). In the combination treatment group, the smallest tumor size and weight, which was extracted on day 20, was found (Figure 6B,C). The biggest mean tumor growth time, tumor growth delay time, and mean growth inhibition rate was also found in the combination treatment group (Table 2). The enhancement ratio for magnolol alone and regorafenib alone was found to be 3.86 and 2.28, respectively. Moreover, the mean tumor growth inhibition rate in the combination group was 78.87, which shows a synergistic inhibition efficacy, as compared to magnolol alone and regorafenib alone (Table 3). The tumor size imaged by micro-CT on day 20 indicated a smaller size with a combination of regorafenib and magnolol than with a single drug administration (Figure 6D). There was no obvious difference between the control and treatment groups in terms of the general toxicity by body weight and normal tissue pathology by H&E (Figure 6E,F). As shown in Figure 6G, anti-apoptosis- and proliferation-related proteins, such as CyclinD1, XIAP, C-FLIP, and MCL-1, were all decreased in the tumor tissues in the combination group. Not only caspase-dependent proteins (cleaved caspase-3, -8, and -9), but also caspase-independent effectors of apoptosis (EndoG and AIF) in tumors were induced by the combination therapy (Figure 6H). As in the in vitro results, the inhibition of the expression of metastasis- and DNA repair-related proteins, such as VEGF-A, MMP9, and MDC-1, was also found in the combination group in vivo (Figure 6I). The expression of these proteins indicated a greater induction and inhibition effect with the combination treatment, compared to the single treatments.

## 4. Discussion

Several tyrosine kinase inhibitors, such as sorafenib, regorafenib, and lenvatinib, have been approved for the treatment of advanced HCC [25,26]. Therefore, the development of the abovementioned tyrosine kinase inhibitors may offer therapeutic benefits for patients with HCC. Previous studies indicated that AKT inactivation was associated with the synergistic anti-HCC effects of magnolol and sorafenib [10]. The major purpose of the present study was to evaluate the anti-HCC effects and mechanism of magnolol combined with regorafenib. The results demonstrated that magnolol significantly increased the regorafenib-inhibited growth of HCC cells in vitro and in vivo (Figure 1C,D).

Vascular endothelial growth factor (VEGF) signaling facilitates tumor angiogenesis, growth and metastasis [27,28]. VEGF-A/VEGF receptor 1(VEGFR1) interaction has been demonstrated to promote cell proliferation and invasion in HCC and CRC cells [29,30]. A high expression of both phospho-VEGFR1/VEGFR2 in resected HCC tissues, before sorafenib treatment, was correlated with a favorable overall survival [29]. Notably, the increased expression of serum VEGF-A has been shown to be an unfavorable prognostic factor that is associated with a poor outcome in patients receiving regorafenib for mCRC [31]. In this study, we verify the abundance or absence of the expression of VEGF-A and determine whether this influences the cytotoxicity of regorafenib in HCC cells. The results showed that the VEGF-A protein significantly diminished the regorafenib-inhibited cell viability, and the transient knockdown of VEGF-A by siRNA effectively sensitized HCC cells to regorafenib (Figure 2C,D). In addition, the protein level of VEGF-A was decreased by siVEGF-A, magnolol, regorafenib, and a combination of magnolol and regorafenib (Figure 1G,H and Figure 2G). The combination group presented a lower protein level of VEGF-A, compared to those treated with magnolol or regorafenib alone, in HCC cells in vitro and in vivo (Figure 1G,H and Figure 6I).

Myeloid cell leukemia 1 (MCL-1), an antiapoptotic B-cell lymphoma 2 (Bcl-2) family protein, blocks intrinsic pathway-initiated apoptosis by interfering with BAX–BAK interaction. The increased expression of MCL-1 has been observed in human cancers and has been associated with the induction of resistance to chemo-radiotherapy and targeting therapy [32,33,34]. The inhibition of MCL-1 expression has been indicated to reverse regorafenib resistance in CRC cells through the restoration of regorafenib-induced apoptosis [34]. Our data showed that siMCL-1 effectively enhanced the regorafenib-mediated inhibition of the growth of HCC cells (Figure 2H,I). We also found that a combination of magnolol and regorafenib may decrease the protein level of MCL-1, compared to the treatments with magnolol or regorafenib alone, in HCC cells in vitro and in vivo (Figure 1G,H and Figure 6G).

In addition to MCL-1 inhibition, DNA damage, and endoplasmic reticulum (ER), stress also triggers apoptosis through mitochondrial caspase-dependent and -independent apoptotic pathways. DNA damage and ER stress induce a loss of mitochondrial membrane potential (ΔΨm), leading to a release of Cytochrome C, ROS, AIF, and EndoG from mitochondria [35,36,37,38]. Both AIF and Endo-G, which are caspase-independent effectors of apoptosis, translocate to the nucleus to promote the formation of chromosomal DNA fragmentation [39]. An accumulation of calcium is characteristic of ER stress-mediated apoptosis [40]. The results demonstrated that magnolol significantly enhanced regorafenib-induced apoptotic events, the percentages of sub-G1 phase, DNA damage, the accumulation of calcium, the loss of △ψm, the generation of ROS, and the expression of cleaved caspase-3 and cleaved PARP-1 in both SK-Hep1 and Hep3B cells (Figure 4A–E and Figure 5). In addition, the combination group upregulated the protein level of BAX and BAK and the nucleus translocation of AIF, unlike the treatments with magnolol or regorafenib alone (Figure 5H–K and Figure 6H).

Both magnolol- and regorafenib-triggered apoptosis were dependent on extrinsic (death receptor) and intrinsic pathways in HCC cells [41,42]. In this study, we found that extrinsic apoptotic signaling, including the activation of death receptors, FAS, FAS-L, and cleaved caspase-8, was obviously increased by the combination of magnolol and regorafenib, compared to the treatments with magnolol or regorafenib alone (Figure 5A–C). According to our data, magnolol enhanced regorafenib-induced apoptosis through extrinsic and intrinsic pathways in HCC cells. Anticancer agent-induced DNA damage can be repaired by an exuberant DNA repair response in cancers. The mediator of DNA damage checkpoint protein-1 (MDC-1), an important mediator of DNA double-strand break repair, can induce the recruitment of DNA repair-associated proteins to DNA injury sites [20,43]. An overexpression of MDC-1 has been shown to mediate tumor resistance to anticancer agents [44,45]. Our data indicated that magnolol combined with regorafenib downregulated the protein level of MDC-1, compared to the treatments with magnolol or regorafenib alone, in HCC in vitro and in vivo (Figure 1G,H and Figure 6I).

## 5. Conclusions

In conclusion, this study found that magnolol, a regorafenib sensitizer, significantly enhanced the therapeutic efficacy of regorafenib in HCC cells in vitro and in vivo. We suggested that the induction of apoptosis and the inhibition of the expression of MCL-1 and VEGF-A are correlated with the anti-HCC efficacy of magnolol combined with regorafenib.

## Figures and Tables

**Table 1:** Combination index data for non-constant combinations.

		Effect (FA)		Combination Index (CI)	
Regorafenib (μM)	Magnolol (μM)	Hep3B	SK-Hep1	Hep3B	SK-Hep1
5	50	0.41	0.34	0.96	1.17
10	50	0.59	0.52	0.83	0.98
15	50	0.73	0.66	0.74	0.90
20	50	0.89	0.8	0.51	0.74

**Table 2:** Mean tumor growth time, delay time, inhibition rate, and enhancement ratio in Hep3B tumor-bearing mice after treatment with magnolol, regorafenib, and a combination of both.

Treatment	MTGT (Day) *	MTGDT (Day) ^#^	MGIR ^$^	ER ^★^
Control	4.25	NA	NA	NA
Magnolol (MAG)	6.44	2.19	1.52	3.86
Regorafenib (REG)	10.93	6.68	2.57	2.28
Combination	24.89	20.64	2.28	--

NA: not available. * Mean tumor growth time (MTGT): the time at which the tumor volume reached 500 mm^3^. ^#^ Mean tumor growth delay time (MTGDT): the mean tumor growth time of the treated group minus that of the control group. ^$^ Mean growth inhibition rate (MGIR): the mean tumor growth time of the treated group/the mean tumor growth time of the sham control group. ^★^ Enhancement ratio (ER): the mean growth inhibition rate of the combination group/the mean growth inhibition rate of the MAG or REG group.

**Table 3:** Mean tumor growth inhibition rate and combination index in Hep3B tumor-bearing mice after treatment with magnolol, regorafenib, and a combination of both.

Treatment	Mean Inhibitory ^*^	Expected Inhibitory ^#^	Combination Index ^$^
Control	--	--	--
Magnolol (MAG)	31.98	--	--
Regorafenib (REG)	58.11	--	--
Combination	78.87	50.37	0.43 (synergistic)

* Mean growth inhibitory rate: 1-mean tumor volume ratio of the treated group/mean tumor volume ratio of the sham control group on the 20th day. ^#^ Expected growth inhibitory rate: the inhibition rates of the combination minus the multiplication of both the MAG and REG inhibition rates. ^$^ Combination index: 1-mean growth inhibitory rate of the combination/1-expected growth inhibitory rate.

## Data Availability

The data generated and analyzed will be made available from the corresponding author on reasonable request.

## Supplementary Materials

The following are available online at https://www.mdpi.com/article/10.3390/cancers13092066/s1, Figure S1. Full Western blot images of Figure 1G,H, Figure S2. Full Western blot images of Figure 2G,J and Figure 5H,I.
