# Supplementary material for: Induction of Apoptosis, Inhibition of MCL-1, and VEGF-A Expression Are Associated with the Anti-Cancer Efficacy of Magnolol Combined with Regorafenib in Hepatocellular Carcinoma
Source: Cancers (Basel). 2021 Apr 25;13(9):2066. doi: 10.3390/cancers13092066 (PMC8123296; doi:10.3390/cancers13092066)
Supplement: Supplementary file 1 [file cancers-13-02066-s001.zip › cancers-1148962-supplementary.pdf]

Supplementary Material: Induction of Apoptosis, Inhibition of MCL-1, and VEGF-A Expression Are Associated with the Anti-Cancer Efficacy of Magnolol Combined with Regorafenib in Hepatocellular Carcinoma

Cheng-Hsien Chen, Fei-Ting Hsu, Wei-Lung Chen and Jiann-Hwa Chen

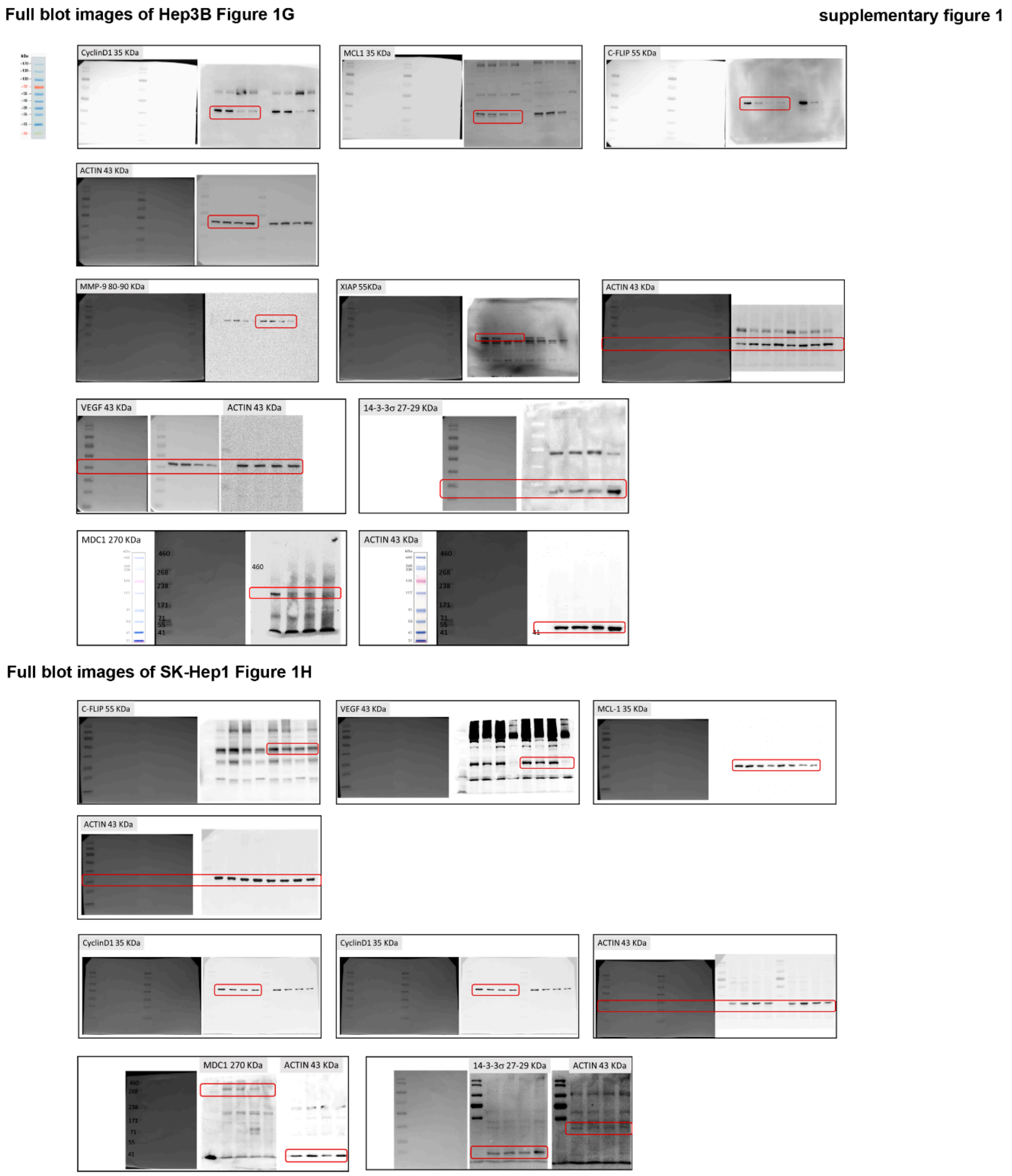

Figure S1. Full Western blot images of Figure 1G,H.

Full blot images of Hep3B and SK-Hep1 Figure 2G-J

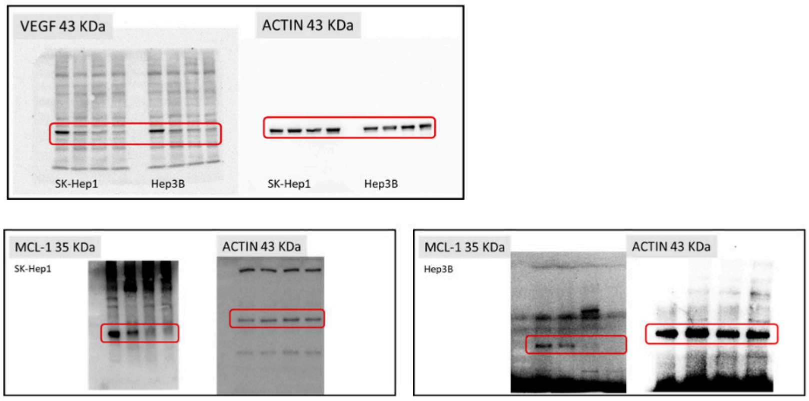

Full blot images of Hep3B and SK-Hep1 Figure 5H-I

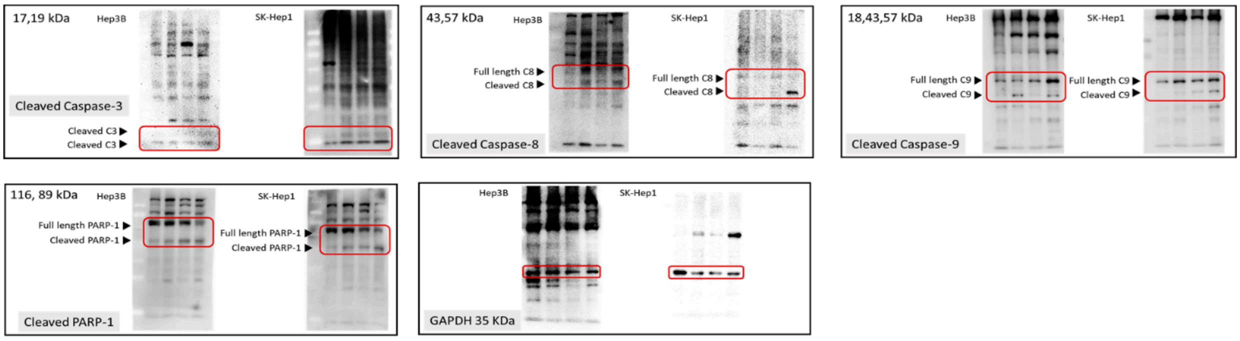

Figure S2. Full Western blot images of Figures 2G,J, 5H,I.
